# Soluble interleukin-2 receptor combined with interleukin-8 is a powerful predictor of future adverse cardiovascular events in patients with acute myocardial infarction

**DOI:** 10.3389/fcvm.2023.1110742

**Published:** 2023-04-17

**Authors:** Kunming Pan, Chenqi Xu, Can Chen, Shuqing Chen, Yuqian Zhang, Xiaoqiang Ding, Xialian Xu, Qianzhou Lv

**Affiliations:** ^1^ Department of Pharmacy, Zhongshan Hospital Fudan University, Shanghai, China; ^2^ Department of Nephrology, Zhongshan Hospital, Fudan University, Shanghai, China; ^3^ Shanghai Institute of Kidney Disease and Dialysis, Shanghai, China; ^4^ Shanghai Key Laboratory of Kidney and Blood Purification, Shanghai, China; ^5^ Shanghai Medical Center of Kidney Disease, Shanghai, China; ^6^ School of Public Health, Tongji Medical College, Huazhong University of Science and Technology, Wuhan, China

**Keywords:** soluble interleukin-2 receptor, interleukin-8, biomarkers, adverse cardiovascular events, myocardial infarction

## Abstract

**Background:**

Little is known about the role of interleukin (IL) in patients with acute myocardial infarction (MI), especially soluble IL-2 receptor (sIL-2R) and IL-8. We aim to evaluate, in MI patients, the predictive value of serum sIL-2R and IL-8 for future major adverse cardiovascular events (MACEs), and compare them with current biomarkers reflecting myocardial inflammation and injury.

**Methods:**

This was a prospective, single-center cohort study. We measured serum concentrations of IL-1β, sIL-2R, IL-6, IL-8 and IL-10. Levels of current biomarkers for predicting MACEs were measured, including high-sensitivity C reactive protein, cardiac troponin T and N-terminal pro-brain natriuretic peptide. Clinical events were collected during 1-year and a median of 2.2 years (long-term) follow-up.

**Results:**

Twenty-four patients (13.8%, 24/173) experienced MACEs during 1-year follow-up and 40 patients (23.1%, 40/173) during long-term follow-up. Of the five interleukins studied, only sIL-2R and IL-8 were independently associated with endpoints during 1-year or long-term follow-up. Patients with high sIL-2R or IL-8 levels (higher than the cutoff value) had a significantly higher risk of MACEs during 1-year (sIL-2R: HR 7.7, 3.3–18.0, *p < *0.001; IL-8: HR 4.8, 2.1–10.7, *p < *0.001) and long-term (sIL-2R: HR 7.7, 3.3–18.0, *p < *0.001; IL-8: HR 4.8, 2.1–10.7, *p < *0.001) follow-up. Receiver operator characteristic curve analysis regarding predictive accuracy for MACEs during 1-year follow-up showed that the area under the curve for sIL-2R, IL-8, sIL-2R combined with IL-8 was 0.66 (0.54–0.79, *p *= 0.011), 0.69 (0.56–0.82, *p < *0.001) and 0.720 (0.59–0.85, *p* < 0.001), whose predictive value were superior to that of current biomarkers. The addition of sIL-2R combined with IL-8 to the existing prediction model resulted in a significant improvement in predictive power (*p *= 0.029), prompting a 20.8% increase in the proportion of correct classifications.

**Conclusions:**

High serum sIL-2R combined with IL-8 levels was significantly associated with MACEs during follow-up in patients with MI, suggesting that sIL-2R combined with IL-8 may be a helpful biomarker for identifying the increased risk of new cardiovascular events. IL-2 and IL-8 would be promising therapeutic targets for anti-inflammatory therapy.

## Introduction

Myocardial infarction (MI) remains a disease with high morbidity and mortality worldwide, although there have been great advances in its treatment (1). Inflammation plays an important role in all phases of atherosclerosis, from initiation to progression to complications and it is still a considerable residual cardiovascular risk factor in patients who received optimal treatment (2). Positive evidence from the CANTOS and COLCOT trials proved that inflammation theory in atherosclerosis is no longer a hypothesis (3). At present, the most successful anti-inflammatory drugs for atherosclerosis are canakinumab, colchicine, etc., whose mechanisms focus on the different levels of the NLRP3 inflammasome pathway, including the downstream pathway of NLRP3: IL-18/IL-1β and the downstream pathway of IL-1: IL-6 (4). Many studies, mainly preclinical, have shown that multiple cytokines and chemokines were induced in the acute inflammatory stage after MI. Studies indicated that interleukin (IL) can predict the long-term adverse clinical events of MI patients, especially IL-1β, IL-6 and IL-10 (5–7). However, studies on the prognostic predictive role of other ILs in patients with MI are still lacking. We wished to explore the predictive value of insufficiently studied ILs outside of the NLRP3 inflammatory pathway for future cardiovascular events in patients with MI, which may be potential therapeutic targets. Based on initial clinical experience and extensive literature reading, we focused our candidate ILs on sIL-2R and IL-8.

IL-2 is a multifunctional cytokine that activates T cells *via* binding to its particular membrane receptor (IL-2 receptor, IL-2R), which is a heterotrimeric receptor composed of IL-2Rα, IL-2Rβ, and IL-2R*γ*. Soluble IL-2R (sIL-2R) is produced by proteolytic cleavage of membrane-bound IL-2Rα (CD25), a part of the high-affinity IL-2R (8). After immunological activation, sIL-2R enters the serum and can be utilized as a biomarker to monitor immune-mediated diseases such as cancer, cardiovascular disease, etc. (9, 10). Whether levels of sIL-2R were increased in MI patients remained controversial. The study by Blum A, et al. revealed that levels of sIL-2R were significantly higher in acute MI (AMI) patients than in controls (11). However, Takeshita S, et al. found that levels of sIL-2R in acute coronary syndrome (ACS) patients were within the range of healthy volunteers (12). To date, the predictive role of sIL-2R in patients with MI was not studied.

IL-8, a typical CXC-type chemokine, is important in promoting the recruitment and activation of neutrophils and monocytes, and may also promote the formation of new blood vessels (13–15). During MI, IL-8 may be involved in promoting the regulation of neutrophil infiltration in ischemia and reperfusion myocardium (16). However, the effect of IL-8 on AMI patients has not been adequately investigated. Several studies have demonstrated elevated IL-8 levels in MI patients compared to those with stable coronary artery disease and controls (15, 17). However, studies on the effect of IL-8 levels on long-term adverse cardiovascular events in MI patients were still lacking (13), and studies in Asian populations were not yet available.

We hypothesized that elevated levels of serum sIL-2R and IL-8 may reflect the inflammation associated with ischemia and reperfusion myocardial injury in patients with MI. We thus designed this prospective study to further evaluate the predictive value of sIL-2R and IL-8 for future adverse cardiovascular endpoints in MI patients, and compare with current biomarkers reflecting myocardial injury and myocardial inflammation (IL-1β, IL-6, IL-10).

## Materials and methods

### Patients

We prospectively enrolled adult AMI patients admitted to the coronary care unit (CCU) between June 1, 2017, and May 31, 2020, at Zhongshan Hospital Fudan University. AMI was defined as when there is acute myocardial injury with clinical evidence of acute myocardial ischemia and with detection of a rise and/or fall of cardiac troponin (cTn) values with at least one value above the 99th percentile URL and at least one of the following: symptoms of myocardial ischemia; new ischemic electrocardiograph changes; development of pathological Q waves; imaging evidence of new loss of viable myocardium or new regional wall motion abnormality in a pattern consistent with an ischemic etiology (18). The exclusion criteria were as follows: inability to provide informed consent, clinical data were not available, and coronary angiography results were not consistent with the diagnosis of myocardial infarction.

### Ethics statement

The studies involving human participants were reviewed and approved by the Ethics Committee of Zhongshan Hospital, Fudan University (Shanghai, China, approval number: B2022–375R). The patients provided written informed consent to participate in this study.

### Laboratory methods

Blood samples were taken after 12 h of overnight fasting on the second day of admission to CCU. All biomarkers were analyzed at the Department of Laboratory Medicine, Zhongshan Hospital, Fudan University. Levels of IL-1β, sIL-2R, IL-6, IL-8 and IL-10 were determined using the chemiluminescence method on automatic chemiluminescence immune analyzer IMMULITE 1,000 (Siemens, Germany). The lower detection limit of IL-1β was 5.0 pg/ml; the upper detection limit of sIL-2R was 7,500 U/ml; the lower detection limit of IL-6 was 2 pg/ml, and the upper detection limit was 1,000 pg/ml; the upper detection limit of IL-8 was >7,500 pg/ml; the lower detection limit of IL-10 was 5 pg/ml, and the upper detection limit was 1,000 pg/ml. When the actual test result is lower than the detection limit, we handle it with the lowest detection limit value. Levels of cardiac troponin T (cTnT) and N-terminal pro-brain natriuretic peptide (NT-proBNP) were determined with electrochemiluminescence on Cobas e 801 (Roche Diagnostics, Germany) and the inter-assay and intra-assay coefficient of variation were <10%. Levels of high sensitivity C reactive protein (hs-CRP) were determined with immunoturbidimetry on Cobas c 702 (Roche Diagnostics, Germany) and the inter-assay and intra-assay coefficient of variation were <5%. Routine biochemical analyses, including total cholesterol, high-density lipoprotein (HDL) cholesterol (mg/dl) and fasting blood sugar were determined by conventional laboratory assays. The diagnostic criteria for diabetes are random blood glucose ≥11.1 mmol/L or fasting blood glucose ≥7.0 mmol/L or oral glucose tolerance test 2-hour blood glucose ≥11.1 mmol/L or glycated hemoglobin A1c ≥ 6.5% (19).

### Clinical follow-up and definition of endpoints

Patients were usually followed up regularly at the outpatient clinic every one to three months after discharge. After all patients were enrolled, the investigators followed up once a year at a centralized time. A composite endpoint of major adverse cardiac events (MACEs) was defined as cardiovascular death, MI, unscheduled revascularization ≥3 months after the index infarction, rehospitalization for heart failure, or stroke. Furthermore, long-term data on composite endpoints were collected after a median of 2.2 years of follow-up.

### Statistical analyses

We used the Kolmogorov-Smirnov test to assess the normality of the variables. Continuous variables were presented as means with standard deviations or medians with interquartile ranges (IQR), and we used independent t-tests or rank-sum tests to compare variables between groups. Qualitative variables are presented as frequencies and corresponding percentages, and we used chi-squared or Fisher's exact tests to compare variables between groups. Associations between interleukins, clinical variables and endpoints were assessed by univariate cox regression analysis. A multivariable cox regression was used to study the association of interleukin with endpoints adjusted for cardiovascular risk factors.

Several risk factors for adverse cardiovascular outcomes in patients with AMI have been identified, and various risk models have been established, such as, Thrombolysis in Myocardial Infarction (TIMI) score (20, 21), and Global Registry of Acute Coronary Events (GRACE) score (22). However, different risk models were developed based on the characteristics of the respective study populations. We thus constructed a “multivariate plus traditional risk factor” model regarding the above risk model, taking into account the population characteristics and sample size of this study. This model included the following factors: heart rate, systolic blood pressure, smoker, diabetes mellitus, hypertension, hypercholesterolemia, coronary artery disease, heart failure, history of stroke, history of percutaneous transluminal coronary intervention (PCI) or coronary artery bypass grafting (CABG), renal insufficiency, anemia, total cholesterol, HDL cholesterol, fasting blood sugar. We also measured biomarkers commonly used to reflect cardiac function, including cTnT, hs-CRP and NT-proBNP. As a result of skewness, the following continuous variables were logarithmically transformed with the natural logarithm: cTnT, hs-CRP, NT-proBNP. We included all covariates with a *p*-value ≤0.05 in univariate analysis and other clinically relevant variables in multivariable models. We used a backward stepwise regression approach to produce the final model. The variance inflation factor (VIF) was used to measure the severity of multicollinearity in regression analysis. The following covariates were included in the model to explore the predictive value of interleukin for endpoint events at 1-year follow-up: age (>65 vs. ≤65 years), heart rate (>100 vs. ≤100 b.p.m.), systolic blood pressure (<100 vs. ≥100 mmHg), renal insufficiency (eGFR <60 vs. ≥60 ml/min), and anemia (yes vs. no). The following covariates were included in the model to explore the predictive value of interleukin for endpoint events at long-term follow-up: age (>65 vs. ≤65 years), heart rate (>100 vs. ≤100 b.p.m.), history of PCI or CABG (yes vs. no), anemia (yes vs. no), renal insufficiency (eGFR <60 vs. ≥60 ml/min), and fasting blood sugar. We used multivariable cox regression to study the association of interleukin with endpoints adjusted for cardiovascular risk factors. sIL-2R and IL-8 remained significantly associated with endpoints after adjusting for cardiovascular risk factors in multivariate Cox regression. We used linear regression analysis to analyze the correlation between sIL-2R and IL-8. We transformed sIL-2R and IL-8 from continuous variables to grouped variables. Cutoff values for the variable groupings were obtained by receiver operator characteristic curve (ROC) analysis, and interleukins were classified as high (greater than the cutoff value) and low (less than or equal to the cutoff value) interleukins levels. Baseline characteristics of the study population stratified by sIL-2R and IL-8 levels were analyzed. Multivariate cox regression was used to analyze the predictive value of high interleukin levels (vs. low interleukin levels) for endpoints. Moreover, we also plotted Kaplan-Meier curves based on the follow-up (years) to estimate the cumulative survival between groups of high and low interleukin levels by log-rank test without adjustment. GRACE score may affect the prognosis of MI patients (22). We compared the predictive value of GRACE score with sIL-2R, IL-8 for prognosis. We assessed the ability of sIL-2R and IL-8 by themselves or in combination with GRACE score to discriminate patients with or without a composite endpoint by the area under the receiver-operating curve. We also evaluated the benefits of adding sIL-2R and IL-8 to the GRACE score using continuous and category-based net reweighting indices (NRI). All *p*-values were two-sided, and a *p*-value ≤0.05 was considered statistically significant. We analyzed the data using SPSS software (version 26.0, IBM Inc., Armonk, NY, United States) and R software, (version 4.2.1; R Core Team).

## Results

### Primary end point

A total of 176 patients with AMI were screened for this study, and 3 cases were excluded (clinical data were not available,1; the final diagnosis was myocarditis, 2). A total of 173 patients were included in this study. The median (IQR) age was 64 (15) years, and 147 (85.0%) were male. Median follow-up was 2.2 years (maximum 5.2 years) (Table 1). A total of 24 patients (13.8% of the total population) experienced MACEs during 1-year follow-up, including 9 cardiovascular deaths (1 history of MI), 13 reinfarctions (3 history of MI), 1 hospitalization for heart failure and 1 stroke. During long-term follow-up (median follow-up, 2.2 years), MACEs occurred in 40 (23.1%) patients, including 12 cardiovascular deaths (1 history of MI, 2 history of strokes, 1 history of stroke and MI), 24 reinfarctions (9 histories of MIs), 1 MI,1 hospitalization for heart failure and 2 strokes (1 history of stroke and MI).

**Table 1:** Clinical and demographic characteristics of patients according to MACEs during 1-year and long-term follow-up.

Factors	All patients *N* = 173	MACEs (1 year) *N* = 24	No MACEs (1 year) *N* = 149	*p*-value	MACEs (long-term) *N* = 40	No MACEs (long-term) *N* = 133	*p*-value
**Demographic Information**							
Age, years	64 (15)	72.5 (16)	63 (14)	<0.001	73.5 (13)	63.0 (15)	<0.001
Male	147 (85.0)	19 (79.2)	128 (85.9)	0.335	30 (75.0)	117 (88.0)	0.437
STEMI (vs. NSTEMI)	93 (53.8)	12 (50.0)	81 (54.4)	0.704	24 (60.0)	69 (51.9)	0.572
Body mass index, kg/m^2^	23.8 (4.5)	22.9 (2.7)	24.1 (4.4)	0.132	23.1 (2.6)	24.2 (4.5)	0.221
**Cardiovascular Risk Factor**							
Heart rate (>100 b.p.m.)	21 (12.1)	6 (25.0)	15 (10.1)	0.028	8 (20.0)	13 (9.8)	0.014
SBP (<100 mmHg)	12 (6.9%)	4 (16.7)	8 (5.4)	0.043	4 (10.0)	8 (6.0)	0.193
Current smoker	79 (45.7)	9 (37.5)	70 (47.0)	0.439	14 (35.0)	65 (48.9)	0.207
Diabetes mellitus	52 (30.1)	9 (37.5)	43 (28.9)	0.380	16 (40.0)	36 (27.1)	0.194
Hypertension	106 (61.3)	16 (66.7)	90 (60.4)	0.542	28 (70.0)	78 (58.6)	0.418
Hypercholesterolemia	16 (9.2)	3 (12.5)	13 (8.7%)	0.600	5 (12.5)	11 (8.3)	0.854
Coronary artery disease	103 (59.5)	15 (62.5)	88 (59.1)	0.779	24 (60.0)	79 (59.4)	0.540
Heart failure	24 (13.9%)	5 (20.8%)	19 (12.8%)	0.235	8 (20.0)	16 (12.0)	0.388
History of PCI or CABG	19 (11.0)	4 (16.7)	15 (10.1)	0.501	10 (25.0)	9 (6.8)	0.027
History of stroke	6 (3.5)	0 (0.0%)	6 (4.0%)	0.506	3 (7.5)	2 (2.3)	0.627
Renal insufficiency (eGFR <60 ml/min)	37 (21.4)	10 (41.7)	27 (18.1)	0.006	17 (42.5)	20 (15.0)	<0.001
Anemia	55 (31.8)	15 (62.5)	40 (26.8)	<0.001	21 (52.5)	34 (25.6)	<0.001
Grace Score	107.8 ± 27.1	130.0 ± 32.9	104.2 ± 24.4	<0.001	125.3 ± 30.4	103.5 ± 24.5	<0.001
Total cholesterol (mg/dl)	4.41 (1.30)	4.69 (1.56)	4.39 (1.34)	0.481	4.385 (1.60)	4.42 (1.24)	0.500
HDL cholesterol (mg/dl)	1.04 (0.34)	1.18 (0.39)	1.02 (0.34)	0.171	1.085 (0.38)	1.02 (0.34)	0.297
Fasting blood sugar	6.05 (2.18)	6.2 (3.0)	6.0 (2.1)	0.018	6.65 (2.4)	5.9 (2.1)	0.037
Biomarkers							
cTnT, ng/ml	2.09 (3.62)	2.09 (5.12)	2.19 (3.58)	0.309	2.15 (4.71)	2.09 (3.58)	0.082
hs-CRP, mg/dl	13.2 (34.6)	33.6 (58.3)	12.6 (32.0)	0.069	23.45 (53.5)	11.4 (30.7)	0.010
NT-proBNP (pg/ml)	797.6 (1630.0)	1699.0 (3266.5)	722.0 (1495.0)	<0.001	1821.0 (2734.3)	677.0 (1074.0)	<0.001
**Interleukin cytokines**							
IL-1β (pg/ml)	5.0 (0.0)	5.0 (0.0)	5.0 (0.0)	0.009	5.0 (0.0)	5.0 (0.0)	0.019
Soluble IL-2 receptor (U/ml)	399.0 (209)	462.5 (626)	390.0 (203)	<0.001	462.5 (425)	377.0 (208)	<0.001
IL-6 (pg/ml)	12.9 (16.8)	21.8 (26.7)	11.8 (16.0)	0.054	20.5 (23.3)	11.5 (13.9)	0.058
IL-8 (pg/ml)	15.0 (20.5)	34.5 (60.7)	13.9 (17.4)	<0.001	21.5 (36.2)	13.9 (17.4)	<0.001
IL-10 (pg/ml)	5.0 (0.0)	5.0 (2.2)	5.0 (0.0)	<0.001	5.0 (0.0)	5.0 (0.0)	0.002

Values are median (interquartile range) or % (n). long-term follow-up, a median follow-up of 2.2 years.

CABG, coronary artery bypass grafting surgery; cTnT, cardiac troponin T; eGFR, estimated glomerular filtration rate; HDL, high-density lipoprotein; hs-CRP, high-sensitivity C-reactive protein; IL, Interleukin; MACEs, major adverse cardiovascular events; MI, myocardial infarction; NSTEMI, non-ST-segment elevation myocardial infarction; NT-proBNP, N-terminal pro-B-type natriuretic peptide; PCI, percutaneous coronary intervention; STEMI, ST-segment elevation myocardial infarction; SBP, Systolic blood pressure.

### Baseline characteristics of patients according to MACEs

Patients who experienced MACEs during 1-year or long-term follow-up tended to be older and more often had cardiovascular risk factors including rapid heart rate (>100 b.p.m.), renal insufficiency (eGFR <60 ml/min), anemia and higher fasting blood sugar, compared with patients without MACEs. Patients who experienced MACEs during 1-year or long-term follow-up had significantly higher GRACE scores and higher levels of NT-proBNP than those without MACEs (Table 1).

### Association between serum interleukin levels and MACEs

In univariable Cox regression analyses, IL-1β, sIL-2R, IL-8 and IL-10 levels were significantly elevated in patients who experienced MACEs during the first year and long-term follow-up compared with patients without (Table 1). After adjustment for clinical characteristics in multivariable Cox regression models, sIL-2R and IL-8 remained significantly elevated in patients who experienced MACEs during the first year (Supplementary Table S1) and long-term follow-up (Supplementary Table S2). Furthermore, multivariable Cox regression analysis showed that among the five interleukins studied, only sIL-2R and IL-8 were independently associated with MACEs during follow-up (Supplementary Table S1, S2). Linear regression analysis showed that serum sIL-2R was positively associated with IL-8 levels (*p *= 0.006), but the model fit was poor (R-squared 0.044) (Supplementary Figure S1). We then convert sIL-2R and IL-8 from continuous variables to grouped variables. Cutoff values were calculated by ROC analysis for dichotomizing IL-8 into high (greater than the cutoff value 32.5 pg/ml) and low levels (less than or equal to 32.5 pg/ml) for 1-year follow-up. The same approach was used to classify sIL-2R as high sIL-2R (greater than the cutoff value 807 U/ml) and low sIL-2R (less than or equal to 807 U/ml).

### Characteristics of patients stratified by levels of sIL-2r and IL-8

Baseline characteristics of the included patients based on levels of sIL-2R (high vs. low) and IL-8 (high vs. low) were shown in Supplementary Table S3. Compared with patients with low sIL-2R levels, patients with high sIL-2R levels tended to be older and had significantly higher total cholesterol, fasting blood sugar, hs-CRP levels, and more often had hypertension, heart rate (>100 b.p.m.), diabetes mellitus, renal insufficiency and anemia. Compared with patients with low IL-8 levels, patients with high IL-8 levels had significantly higher fasting blood sugar and NT-proBNP levels, and more often had coronary artery disease.

### Association between high levels of sIL-2r and MACEs

**Figure 1:**
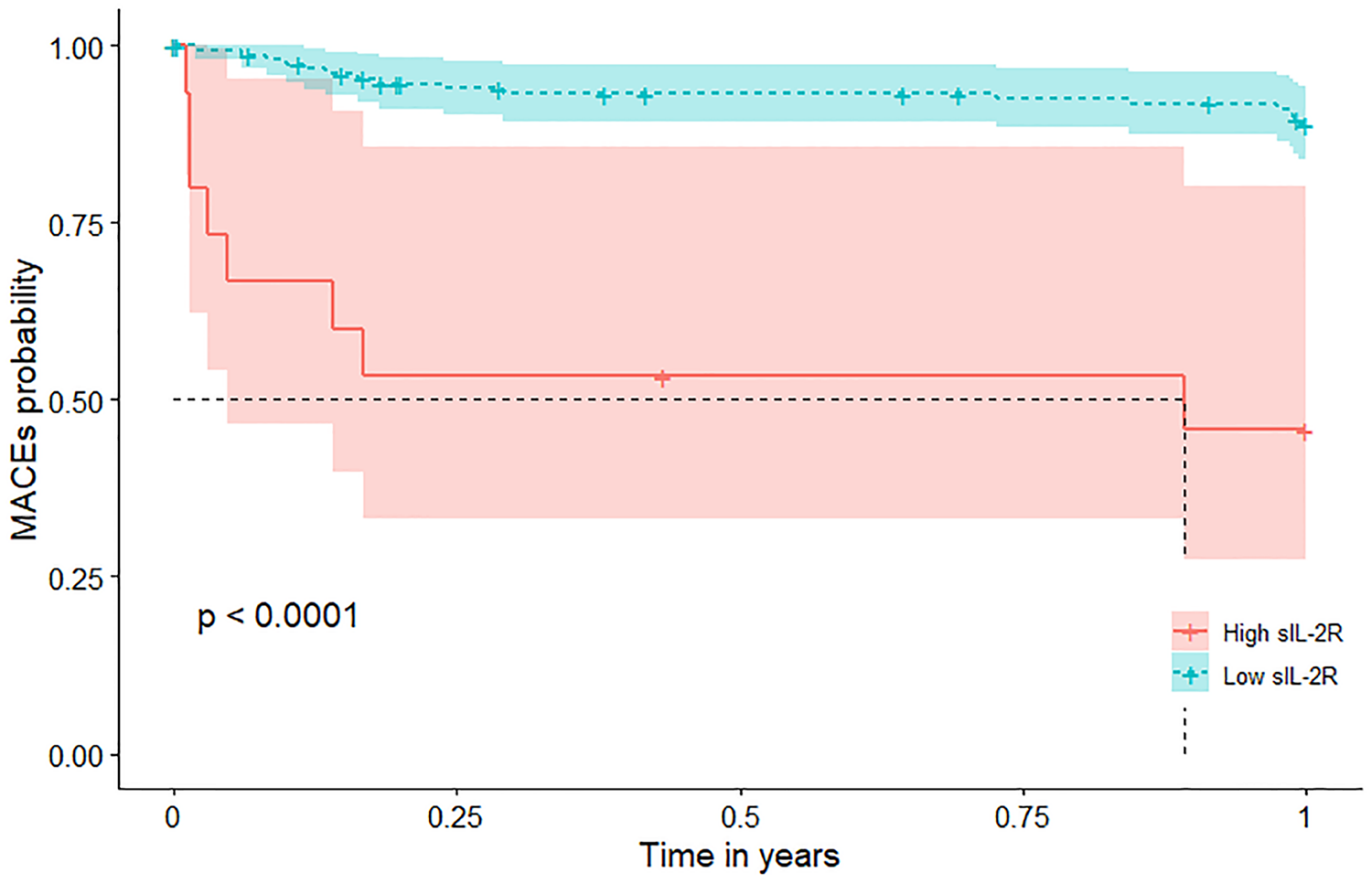
MACEs According to sIL-2R Levels in patients with MI during 1-year follow-up. Kaplan-Meier plots of MACEs during 1-year follow-up according to high (greater than the cutoff value 807 U/ml) or low sIL-2R (less than or equal to 807 U/ml). sIL-2R was measured after a 12-h overnight fast on the second day of admission of CCU. CCU, coronary care unit; IL, interleukin; MACEs, major adverse cardiovascular events; MI, myocardial infarction; sIL-2R, soluble IL-2 receptor.

Patients with high sIL-2R levels (higher than the cutoff value) had a significantly higher risk of MACEs during 1-year follow-up (Figure 1) with a hazard ratio (HR) of 7.7 (95% CI: 3.3–18.0; *p* < 0.001) (Table 2). This association remained significant after adjustment for relevant significant cardiovascular risk factors (adjusted HR: 3.5; 95% CI: 1.4–9.0; *p* < 0.001) and all cardiovascular risk factors (adjusted HR: 3.7; 95% CI: 1.2–11.9; *p *= 0.028) (Table 2). Also, patients with high sIL-2R levels had a significantly higher risk of MACEs during long-term follow-up (HR: 2.9; 95% CI: 1.4–5.7; *p *= 0.003), which remained significant after adjustment for different cardiovascular risk factors (Supplementary Figure S2 and Table S4). The results of VIF between the factors were all <2 in model 1 and <3 in model 2.

**Table 2:** Univariable and multivariable HR of high sIL-2R for MACEs at 1-year follow-up.

	HR	95% Cl.	*p*-value
**Univariable analysis**			
High sIL-2R (>Cutoff value)^a^	7.7	3.3–18.0	<0.001
Age (>65 years)	5.2	1.9–13.9	<0.001
Heart rate (>100 b.p.m.)	2.8	1.1–7.1	0.028
Systolic blood pressure (<100 mmHg)	3.0	1.04–8.89	0.043
Known renal insufficiency (eGFR <60 ml/min)	3.1	1.4–7.0	0.006
Anemia	4.2	1.9–9.7	<0.001
**Multivariable analysis**			
Model 1^b^			
High sIL-2R (>Cutoff value)^a^	3.5	1.4–9.0	<0.001
Age (>65 years)	3.6	1.2–11.0	0.024
Systolic blood pressure (<100 mmHg)	6.6	2.0–21.6	0.002
Anemia	2.5	1.001–6.210	0.0498
Model 2^c^			
High sIL-2R (>Cutoff value)	3.7	1.2–11.9	0.028
Age (>65 years)	4.4	1.2–16.4	0.027
Systolic blood pressure (<100 mmHg)	6.6	1.7–25.9	0.007
Total cholesterol (mg/dl)	1.6	1.2–2.3	0.004
Anemia	3.2	1.1–9.4	0.036

CABG, coronary artery bypass grafting surgery; HDL, high-density lipoprotein; IL, interleukin; PCI, percutaneous coronary intervention; sIL-2R, soluble IL-2 receptor.

^a^ High sIL-2R defined as sIL-2R levels greater than the cutoff value 807 U/ml.

^b^ Model 1 adjusted for age (>65 years), heart rate (>100 b.p.m.), systolic blood pressure (<100 mmHg), Renal insufficiency (eGFR <60 ml/min) and anemia.

^c^ Model 2: model 1 +sex, body mass index, current smoker, diabetes mellitus, hypertension, hypercholesterolemia, coronary artery disease, heart failure, history of PCI or CABG, history of stroke, total cholesterol, HDL cholesterol, and fasting blood sugar.

### Association between high levels of IL-8 and MACEs

**Figure 2:**
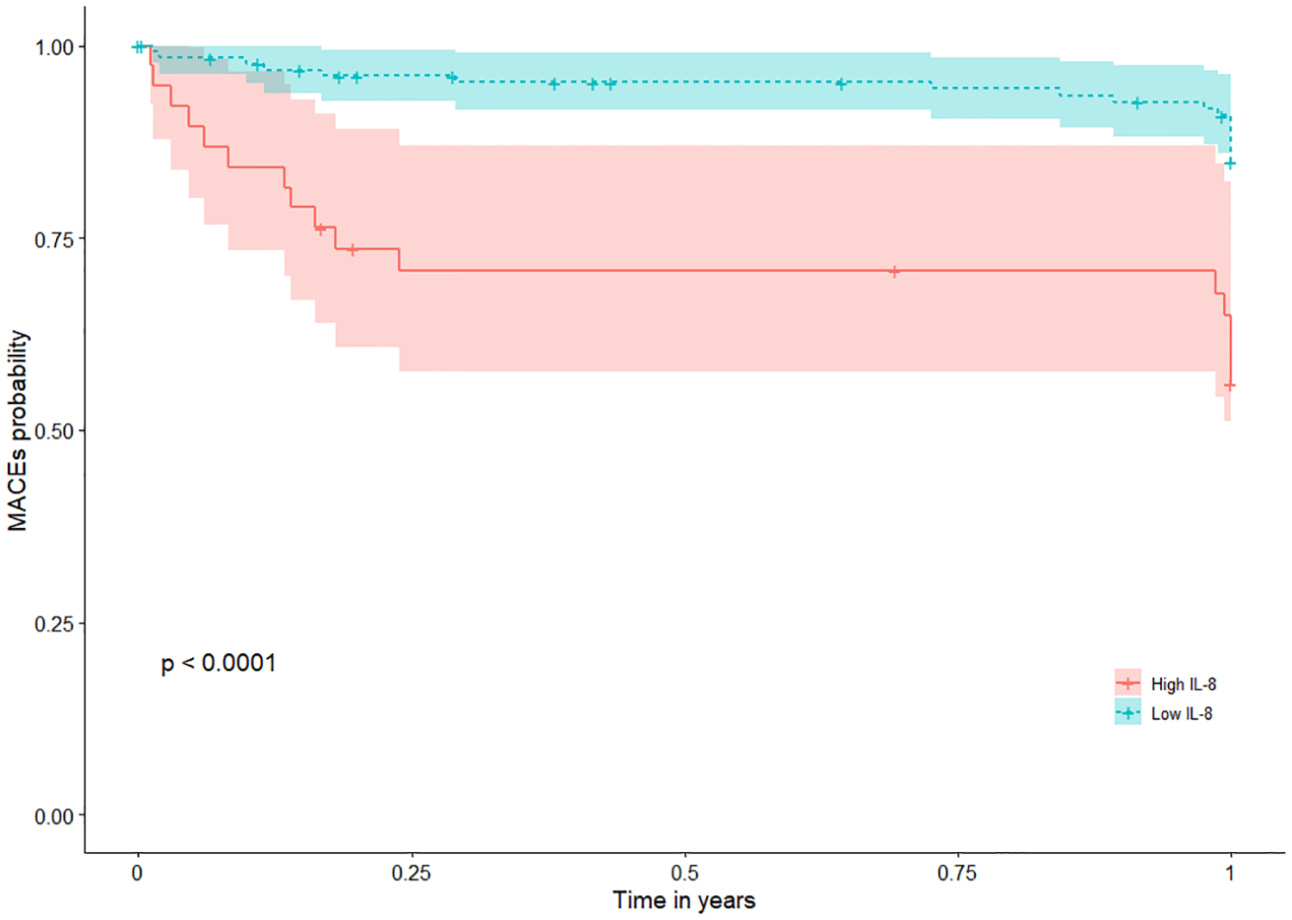
MACEs according to IL-8 Levels in patients with MI during 1-year follow-up. Kaplan-Meier plots of MACEs during 1-year follow-up according to high (greater than the cutoff value 32.5 pg/ml) or low IL-8 (less than or equal to 32.5 pg/ml). IL-8 was measured after a 12-h overnight fast on the second day of admission of CCU. CCU, coronary care unit; IL, interleukin; MACEs, major adverse cardiovascular events; MI, myocardial infarction; sIL-2R, soluble IL-2 receptor.

Patients with high IL-8 levels (higher than the cutoff value) had a significantly higher risk of MACEs during 1-year follow-up (Figure 2) with an HR of 4.8 (95% CI: 2.1–10.7; *p* < 0.001) (Table 3). This association remained significant after adjustment for relevant significant cardiovascular risk factors (adjusted HR: 4.4; 95% CI: 1.9–9.9; *p* < 0.001) and all cardiovascular risk factors (adjusted HR: 6.2; 95% CI: 2.2–17.5; *p *= 0.006) (Table 3). Also, patients with high IL-8 levels had a significantly higher risk of MACEs during long-term follow-up (HR: 3.3; 95% CI: 1.8–6.4; *p* < 0.001), which remained significant after adjustment for different cardiovascular risk factors (Supplementary Figure S3 and Table S5). The results of VIF between the factors were all <2 in model 1 and <3 in model 2.

**Table 3:** Univariable and multivariable HR of high IL-8 for MACEs at 1 year follow-up.

	HR	95% Cl.	*p*-value
**Univariable analysis**			
High IL-8 (>Cutoff value)^a^	4.8	2.1–10.7	<0.001
Age (>65 years)	5.2	1.9–13.9	<0.001
Heart rate (>100 b.p.m.)	2.8	1.1–7.1	0.028
Systolic blood pressure (<100 mmHg)	3.0	1.04–8.89	0.043
Known renal insufficiency (eGFR <60 ml/min)	3.1	1.4–70	0.006
Anemia	4.2	1.9–9.7	<0.001
**Multivariable analysis**			
Model 1^b^			
High IL-8 (>Cutoff value)^a^	4.4	1.9–9.9	<0.001
Age (>65 years)	4.1	1.5–11.4	0.008
Systolic blood pressure (<100 mmHg)	5.8	1.8–19.1	0.004
Anemia	3.5	1.4–8.6	0.006
Model 2^c^			
High IL-8	6.2	2.2–17.5	<0.001
Age (>65 years)	5.7	1.5–21.8	0.011
Current smoker	0.3	0.11–1.03	0.057
Systolic blood pressure (<100 mmHg)	6.2	1.6–24.7	0.010
Total cholesterol (mg/dl)	1.5	1.1–2.1	0.020
Anemia	5.2	1.8–14.9	0.002

CABG, coronary artery bypass grafting surgery; HDL, high-density lipoprotein; IL, interleukin; PCI, percutaneous coronary intervention.

^a^ High IL-8 defined as IL-8 levels greater than the cutoff value 32.5 pg/ml.

^b^ Model 1 adjusted for age (>65 years), heart rate (>100 b.p.m.), systolic blood pressure (<100 mmHg), Renal insufficiency (eGFR <60 ml/min) and anemia.

^c^ Model 2: model 1 + sex, body mass index, current smoker, diabetes mellitus, hypertension, hypercholesterolemia, coronary artery disease, heart failure, history of PCI or CABG, history of stroke, total cholesterol, HDL cholesterol, and fasting blood sugar.

### Predictive value of sIL-2r combined with IL-8 levels for MACEs

**Figure 3:**
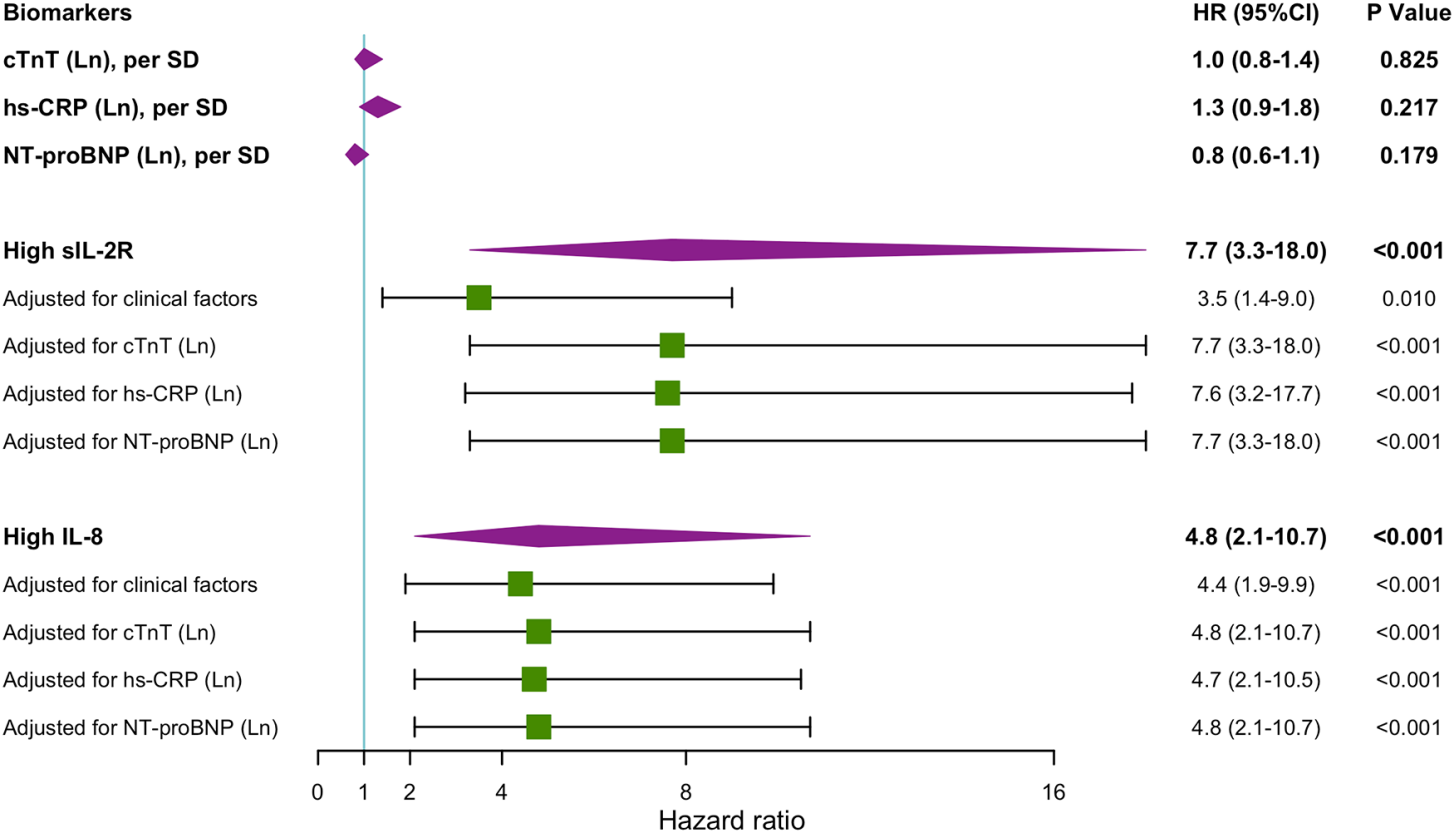
Hazard ratios for MACEs 1-year follow-up. ﻿Unadjusted and adjusted hazard ratios (HRs) obtained by Cox regression analyses for MACEs during 1-year follow-up when having high sIL-2R (greater than the cutoff value 807 U/ml) and high IL-8 (greater than the cutoff value 32.5 pg/ml) levels during hospitalization. sIL-2R and IL-8 was measured after a 12-h overnight fast on the second day of admission of CCU. clinical factors include: heart rate, systolic blood pressure, smoker, diabetes mellitus, hypertension, hypercholesterolemia, coronary artery disease, heart failure, history of PCI or CABG, history of stroke, renal insufficiency, anemia, total cholesterol, HDL cholesterol, fasting blood sugar. CABG, coronary artery bypass grafting; CCU, coronary care unit, cTnT, cardiac troponin T; hs-CRP, high sensitivity C reactive protein; IL, interleukin; Ln, natural logarithm; MACEs, major adverse cardiovascular events; MI, myocardial infarction; NT-proBNP, N-terminal pro-brain natriuretic peptide; PCI, percutaneous transluminal coronary intervention; SD, standard deviation; sIL-2R, soluble IL-2 receptor.

**Figure 4:**
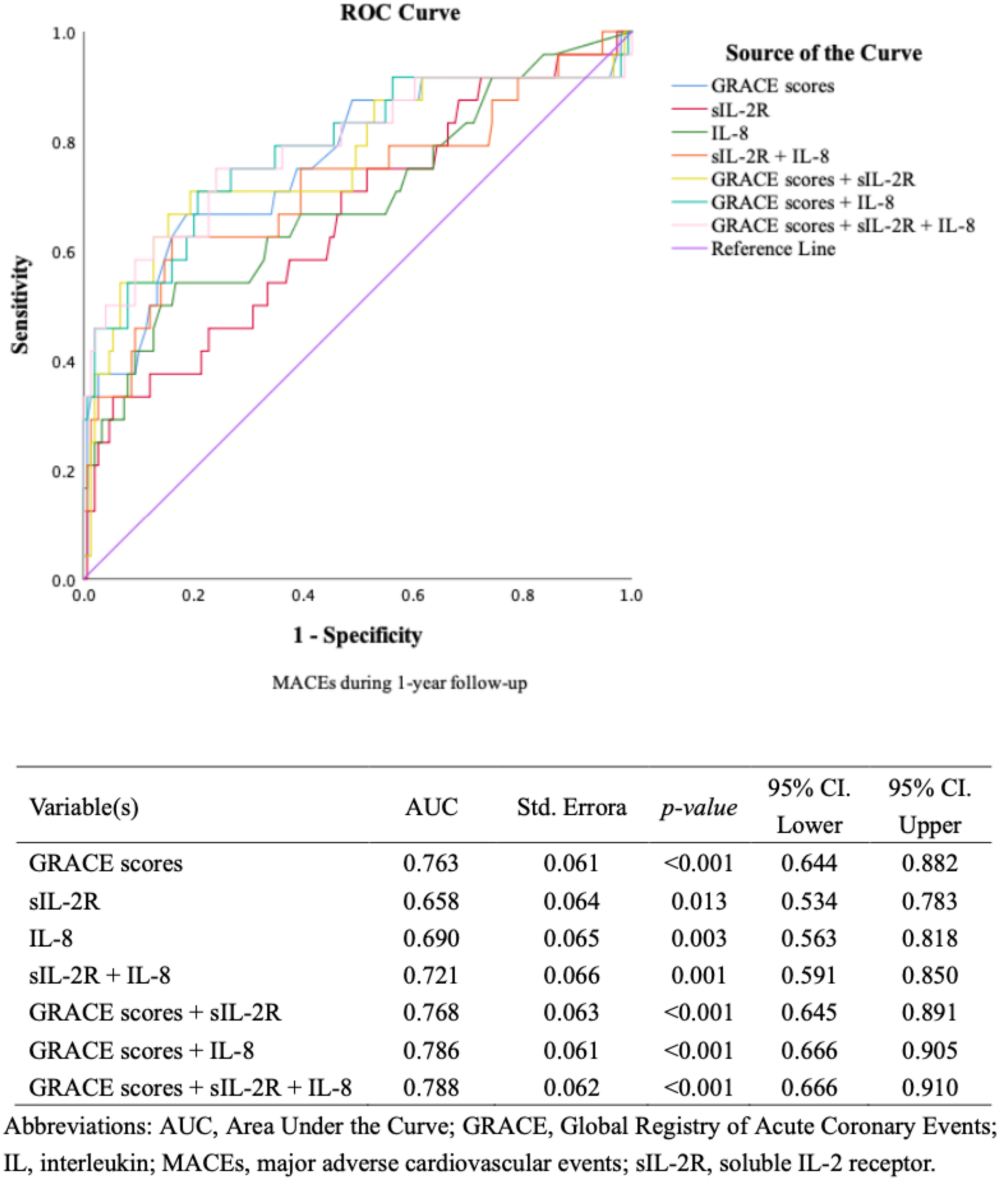
ROC curves for discriminating between patients with or without MACEs (IL, GRACE). ROC curves for discriminating between patients with or without MACEs during 1-year follow-up according to different biomarkers.

Multivariable Cox regression analysis models including cardiovascular risk factors, cTnT levels, hs-CRP levels, and NT-proBNP levels showed that high levels of sIL-2R and IL-8 remained associated with an increased risk of MACEs during the first year's follow-up (Figure 3), as well as during the long-term follow-up (Supplementary Figure S4). ROC analysis regarding predictive accuracy for MACEs during the first-year follow-up showed that the area under the curve (AUC) was 0.763 for GRACE scores (0.64–0.88, *p* < 0.001), 0.66 (0.54–0.79, *p *= 0.011) for sIL-2R levels and 0.69 (0.56–0.82, *p* < 0.001) for IL-8 levels (Figure 4). ROC analysis did not find any significant predictive value of cTnT levels, hs-CRP levels, and NT-proBNP levels for MACEs during the first-year follow-up (Supplementary Figure S5). The sIL-2R combined with IL-8 levels had further better predictive value for MACEs than sIL-2R, IL-8 alone during the first-year follow-up, with an AUC of 0.720 (0.59–0.85, *p* < 0.001) (Figure 4).

The GRACE score combined with sIL-2R, IL-8 and sIL-2 combined with IL-8 further improved the predictive power for MACEs during the first-year follow-up, with AUCs of 0.768 (0.645–0.891, *p* < 0.001), 0.786 (0.666–0.905, *p* < 0.001) and 0.788 (0.666–0.910, *p* < 0.001), respectively. Adding sIL-2R, IL-8, sIL-2R combined with IL-8 to GRACE score for predicting MACEs during the first year's follow-up reclassified a large proportion of patients to the correct risk stratum. The NRI values of adding sIL-2R, IL-8, and sIL-2R combined with IL-8 to the GRACE score were 0.0766 (*p* = 0.355), 0.167 (*p* = 0.129), and 0.208 (*p* = 0.029) respectively (Figure 4). The addition of sIL-2R combined with IL8 to the GRACE score led to a significant improvement in the predictive power of the new model, contributing to a 20.8% increase in the proportion of correct classifications. We also compared typical model performance metrics between the different models, and the results for Akaike information criterion (AIC) and Bayesian information criterion (BIC) were shown in the Supplementary Table S6.

## Discussion

The main findings of this study were that high serum sIL-2R combined with IL-8 levels has significant predictive value for MACEs in patients with MI during 1-year and a median follow-up of 2.2 years, after adjusting for clinical risk factors and current biomarkers reflecting myocardial inflammation and injury. To the best of our knowledge, this was the first study to simultaneously assess the predictive value of sIL-2R and IL-8 on future adverse cardiovascular events in patients with MI.

To our knowledge, this was the first study to examine the association between levels of sIL-2R in serum and MACEs in MI patients. sIL-2R was low in resting T cells, but was rapidly increased upon T cell activation. Therefore, sIL-2R levels were considered a marker of immune system activation (9, 10). Our study showed that high sIL-2R levels were associated with future MACEs, which remained significant after adjusting for significant risk factors and all measured baseline factors (the most simple and reliable analysis) separately. A study evaluated the association between sIL-2R and reinfarction within 7 days in patients with new MI and found significantly higher slL-2R levels in patients with reinfarction compared with those without reinfarction (11). It has been suggested that high sIL-2R reflects not only local inflammatory activation at the site of myocardial infarction but also a reflection of systemic inflammatory activity (23). Inflammation has been proven to increase cardiovascular risk in patients with MI, and anti-inflammatory treatments such as colchicine can reduce cardiovascular risk by decreasing inflammation levels (24).

Previous studies have shown that levels of IL-8 were elevated in patients with ACS compared to those with chronic stable angina or healthy controls (15, 17). IL-8 was suggested to promote plaque rupture and thrombosis, resulting in MI events through multiple mechanisms (25), such as by enhancing the endothelial adhesiveness for monocytes, by acting as a mitogenic and chemoattractant on smooth muscle cells and by modulating angiogenesis inside the atherosclerotic plaque (26–28). However, there were also views that inflammation immediately after an MI was largely a response to the myocardial injury and a result of the stress and hypoperfusion that may occur, rather than a direct cause of clinical events (29). To date, there were still a lack of studies specifically examining the prognostic utility of IL-8 levels in MI patients. We showed that high IL-8 levels were related to increased risk of future MACEs in MI patients, independent of significant risk factors and all measured baseline factors (the most simple and reliable analysis) separately. Our results were in line with the previous study in ST-segment elevation MI (STEMI) patients demonstrating that high serum IL-8 levels in AMI and a stable phase 4 months post-MI were both associated with MACEs, however, its findings did not apply to a large number of patients with Non-STEMI (13). Another study conducted at a Veterans Administration Medical Center demonstrated that high baseline serum IL-8 levels were independently associated with long-term all-cause mortality in ACS patients, however, the results could not be extrapolated to the women population because the study population was exclusively males (25). It remains unclear how IL-8 contributes to adverse cardiovascular events in patients with MI. Previous studies suggested that IL-8 may down-regulate the expression of tissue inhibitors of metalloproteinase-1 in macrophages and increase metalloproteinase activity, leading to the destruction of existing atherosclerotic plaques (25, 30). In addition, several case-control studies and bioinformatic analyses showed that ACS patients were more likely to have IL-8 gene polymorphisms associated with increased levels of IL-8 compared to controls (31–33). We agreed that increased IL-8 levels reflected exaggerated inflammatory response and that prolonged and excessive IL-8 release contributed to the destabilization of atherosclerotic plaques and raised the possibility of additional atherothrombotic events.

The present study identified sIL-2R combined with IL-8 as a significant independent prognostic factor for future MACEs in patients with MI. In addition, sIL-2R and IL-8 were found to be better prognostic factors than current myocardial markers including cTnT, hs-CRP, NT-proBNP and other interleukin indicators including IL-1β, IL-6 and IL-10. This suggests that sIL-2R and IL-8 may reflect unfavorable aspects beyond myocardial injury, heart failure, etc (13). ROC curve analysis showed that the AUC for sIL-2R and IL-8 was 0.66 and 0.69, respectively, both of which were higher than the current predictors. Encouragingly, the AUC for sIL-2R combined with IL-8 was 0.72, which was a relatively satisfactory result. In addition, sIL-2R combined with IL-8 could further increase the predictive effect of existing models. We took the GRACE score as an example, which is a complex model with good predictive effect (AUC is 0.788). The addition of sIL-2R combined with IL8 to the GRACE score led to a significant improvement in the predictive power of the new model, contributing to a 20.8% increase in the proportion of correct classifications. Our study integrated sIL-2R combined with IL-8 as joint predictor for the first time and found that their predictive value for the prognosis of MI patients was further enhanced. Linear regression analysis suggested a possible positive correlation between sIL-2R and IL-8, and we speculated that the potential mechanism is that both sIL-2R and IL-8 were implicated in the inflammatory response to myocardial infarction. However, due to poor model fit, we expect a larger sample size of tailored studies to explore the relationship between sIL-2R and IL-8. We agreed that sIL-2R combined with IL-8 ha great potential as a powerful biomarker for predicting MACEs in patients with MI and can be applied in the clinic to further improve the risk stratification post-MI.

Cardiovascular disease remains the main cause of death globally, and novel treatment strategies and improvements to existing therapies are required (34). Targeting inflammation is an emerging anti-arteriosclerotic therapy (35). In recent clinical trials, novel IL-1β and IL-6 antagonists have shown promising cardiac benefits (36). However, numerous clinical trials have ultimately failed, which we speculate is related to the complexity of inflammatory pathways and insufficient target specificity (29). Our study suggested that compared with IL1β, IL-6 and IL-10, sIL-2R and IL-8 may be more specific for patients with MI and thus may be promising new therapeutic targets. Studies have shown that injection of recombinant human IL-2 positively influenced cardiac function by improving angiogenesis through a process involving natural killer cells (37, 38). Using low-dose IL-2 to promote the polarization of anti-atherosclerotic regulatory T (Treg) cells is studied in the ongoing (IVORY, NCT04241601) and completed trials (LILACS, NCT03113773, No Results Posted) (39). Trials using antibodies against IL-8 for treatment in cancer patients are under investigation (NCT02536469) (40).

Our study had several limitations. Firstly, this was a prospective observational study in nature, thus the results do not prove a causal relationship involving IL-8, sIL-2R and adverse clinical outcomes. Secondly, our sample size was relatively small, and we will confirm these findings in a larger, multicenter prospective study. Thirdly, the lower limit of detection for IL-1β and IL-10 was 5 pg/ml, which we believe was too high. There were large numbers of patients whose actual test results were below the lower limit of detection. The analysis related to IL-1β and IL-10 may not be accurate enough, so the conclusions related to IL-1β and IL-10 in this study need to be viewed with caution. This study had one strength. The blood samples were collected and then tested by the Laboratory Department of Zhongshan Hospital, which did not undergo frozen storage. This helps to avoid potential storage errors due to sampling stability. Also, because the samples were tested by the hospital, the study results were more amenable to clinical replication.

## Conclusion

In conclusion, this study reported for the first time that high levels of serum sIL-2R were associated with adverse cardiovascular events in patients with MI. This study also showed high levels of serum IL-8 were associated with adverse cardiovascular events in MI patients. Our results suggest that sIL-2R combined with IL-8 may be a helpful biomarker for identifying the increased risk of new cardiovascular events in MI patients and IL-2 and IL-8 would be promising therapeutic targets for anti-inflammatory therapy. Larger, multicenter, prospective studies are needed to assess the association of sIL-2R and IL-8 with future cardiovascular events.

## Data Availability

The original contributions presented in the study are included in the article/supplementary material, further inquiries can be directed to the corresponding authors.
