# Supplementary material for: Soluble interleukin-2 receptor combined with interleukin-8 is a powerful predictor of future adverse cardiovascular events in patients with acute myocardial infarction
Source: Front Cardiovasc Med. 2023 Apr 17;10:1110742. doi: 10.3389/fcvm.2023.1110742 (PMC10150071; doi:10.3389/fcvm.2023.1110742)
Supplement: Supplementary file 1 [file Table1.docx]

Supplementary Table 1. Association between interleukin levels and MACEs at 1-year follow-up in multivariable Cox regression analyses

|  | Independent Variable | HR | 95%Cl. | *p* Value |
| --- | --- | --- | --- | --- |
| Soluble interleukin-2 receptor | Enter regression | | | |
|  | Soluble interleukin-2 receptor | 1.000658 | 1.00009 - 1.00123 | 0.023 |
|  | Age (＞65yrs) | 3.3 | 1.1 - 10.4 | 0.041 |
|  | Heart rate (＞100b.p.m.) | 1.7 | 0.6 - 5.0 | 0.340 |
|  | Systolic blood pressure (＜100 mmHg) | 6.9 | 2.1 - 23.0 | 0.002 |
|  | Renal insufficiency (eGFR <60 mL/min) | 1.3 | 0.5 - 3.5 | 0.631 |
|  | Anemia | 3.2 | 1.3 - 7.8 | 0.010 |
|  | Backward stepwise regression | | | |
|  | Soluble interleukin-2 receptor | 1.000654 | 1.00012 - 1.00119 | 0.016 |
|  | Age (＞65yrs) | 4.0 | 1.3 - 12.0 | 0.014 |
|  | Systolic blood pressure (＜100 mmHg) | 7.3 | 2.2 - 24.3 | <0.001 |
|  | Anemia | 3.2 | 1.3 - 7.8 | 0.009 |
| Interleukin-8 | Enter regression | | | |
|  | Interleukin-8 | 1.014 | 1.005 - 1.023 | 0.002 |
|  | Age (＞65yrs) | 2.8 | 0.9 - 8.6 | 0.074 |
|  | Heart rate (＞100b.p.m.) | 0.9 | 0.3 - 3.1 | 0.929 |
|  | Systolic blood pressure (＜100 mmHg) | 3.7 | 1.0 - 14.4 | 0.055 |
|  | Renal insufficiency (eGFR <60 mL/min) | 1.7 | 0.7 - 4.4 | 0.251 |
|  | Anemia | 3.2 | 1.3 - 8.2 | 0.013 |
|  | Backward stepwise regression | | | |
|  | Interleukin-8 | 1.017 | 1.010 - 1.025 | <0.001 |
|  | Age (＞65yrs) | 2.9 | 1.0 - 8.5 | 0.049 |
|  | Anemia | 2.7 | 1.1 - 6.7 | 0.026 |
| Interleukin-1β | Enter regression | | | |
|  | Interleukin-1β | 1.032 | 0.942 - 1.130 | 0.504 |
|  | Age (＞65yrs) | 3.9 | 1.3 - 11.7 | 0.017 |
|  | Heart rate (＞100b.p.m.) | 1.6 | 0.5 - 5.1 | 0.412 |
|  | Systolic blood pressure (＜100 mmHg) | 5.9 | 1.8 - 19.4 | 0.003 |
|  | Renal insufficiency (eGFR <60 mL/min) | 1.3 | 0.5 - 3.5 | 0.630 |
|  | Anemia | 3.1 | 1.3 - 7.6 | 0.011 |
|  | Backward stepwise regression | | | |
|  | Interleukin-1β | / | / | 0.159 |
|  | Age (＞65yrs) | 4.8 | 1.7 - 13.9 | 0.004 |
|  | Systolic blood pressure (＜100 mmHg) | 6.4 | 2 - 20.9 | 0.002 |
|  | Anemia | 3.1 | 1.3 - 7.5 | 0.010 |
| Interleukin-6 | Enter regression | | | |
|  | Interleukin-6 | 1.002 | 0.996 - 1.008 | 0.570 |
|  | Age (＞65yrs) | 3.8 | 1.2 - 11.6 | 0.022 |
|  | Heart rate (＞100b.p.m.) | 1.7 | 0.6 - 5.2 | 0.322 |
|  | Systolic blood pressure (＜100 mmHg) | 6.3 | 1.9 - 20.5 | 0.002 |
|  | Renal insufficiency (eGFR <60 mL/min) | 1.4 | 0.5 - 3.7 | 0.551 |
|  | Anemia | 3.1 | 1.3 - 7.6 | 0.012 |
|  | Backward stepwise regression | | | |
|  | Interleukin-6 |  |  | 0.534 |
|  | Age (＞65yrs) | 4.8 | 1.7 - 13.9 | 0.004 |
|  | Systolic blood pressure (＜100 mmHg) | 6.4 | 2.0 - 20.9 | 0.002 |
|  | Anemia | 3.1 | 1.3 - 7.5 | 0.010 |
| Interleukin-10 | Enter regression | | | |
|  | Interleukin-10 | 1.024 | 0.981 - 1.069 | 0.275 |
|  | Age (＞65yrs) | 3.9 | 1.3 - 11.9 | 0.016 |
|  | Heart rate (＞100b.p.m.) | 1.4 | 0.4 - 4.8 | 0.546 |
|  | Systolic blood pressure (＜100 mmHg) | 6.2 | 1.9 - 20.1 | 0.003 |
|  | Renal insufficiency (eGFR <60 mL/min) | 1.3 | 0.5 - 3.5 | 0.605 |
|  | Anemia | 3.0 | 1.2 - 7.3 | 0.017 |
|  | Backward stepwise regression | | | |
|  | Interleukin-10 | 1.036 | 0.998 - 1.075 | 0.065 |
|  | Age (＞65yrs) | 4.5 | 1.5 - 13.0 | 0.006 |
|  | Systolic blood pressure (＜100 mmHg) | 6.3 | 1.9 - 20.6 | 0.002 |
|  | Anemia | 3.0 | 1.2 - 7.1 | 0.018 |
|  | | | | |
| Interleukin | Independent Variable | HR | 95% Cl. | *p* Value |
|  | Enter regression | | | |
|  | Interleukin-1β | 0.9523 | 0.8454 - 1.0726 | 0.215 |
|  | Soluble interleukin-2 receptor | 1.1462 | 0.9237 - 1.4223 | 0.004 |
|  | Interleukin-6 | 1.0007 | 1.0002 - 1.0012 | 0.192 |
|  | Interleukin-8 | 1.0043 | 0.9978 - 1.0109 | <0.001 |
|  | Interleukin-10 | 1.0192 | 1.0098 - 1.0286 | 0.420 |
|  | Backward stepwise regression | | | |
|  | Soluble interleukin-2 receptor | 1.0007 | 1.0002 - 1.0012 | 0.003 |
|  | Interleukin-8 | 1.0179 | 1.0109 - 1.0248 | <0.001 |

long-term follow-up, a median follow-up of 2.2 years. Abbreviations: CABG, coronary artery bypass grafting surgery; PCI, percutaneous coronary intervention.
