# Supplementary material for: Soluble interleukin-2 receptor combined with interleukin-8 is a powerful predictor of future adverse cardiovascular events in patients with acute myocardial infarction
Source: Front Cardiovasc Med. 2023 Apr 17;10:1110742. doi: 10.3389/fcvm.2023.1110742 (PMC10150071; doi:10.3389/fcvm.2023.1110742)
Supplement: Supplementary file 2 [file Table2.docx]

Supplementary Table 2. Association between interleukin levels and MACEs at long-term follow-up in multivariable Cox regression analyses

| Interleukin | Independent Variable | HR | 95%Cl. | *p* Value |
| --- | --- | --- | --- | --- |
| Soluble interleukin-2 receptor | Enter regression | | | |
|  | Soluble interleukin-2 receptor | 1.000 | (1.000 - 1.001) | 0.139 |
|  | Age (＞65yrs) | 1.6 | (0.672 - 4.029) | 0.275 |
|  | History of PCI or CABG | 2.9 | (1.327 - 6.443) | 0.008 |
|  | Heart rate (＞100b.p.m.) | 1.0 | (0.418 - 2.557) | 0.943 |
|  | Fasting blood-glucose | 1.1 | (0.929 - 1.2) | 0.406 |
|  | Renal insufficiency (eGFR <60 mL/min) | 2.8 | (1.287 - 6.058) | 0.009 |
|  | Anemia | 2.0 | (0.922 - 4.172) | 0.080 |
|  | Backward stepwise regression | | | |
|  | Soluble interleukin-2 receptor | 1.000 | (1.000 - 1.001) | 0.035 |
|  | History of PCI or CABG | 3.1 | (1.4 - 6.8) | 0.004 |
|  | Renal insufficiency (eGFR <60 mL/min) | 3.3 | (1.7 - 6.6) | <0.001 |
|  | Anemia | 2.4 | (1.2 - 4.8) | 0.012 |
| Interleukin-8 | Enter regression. | | | |
|  | Interleukin-8 | 1.018 | (1.008 - 1.027) | <0.001 |
|  | Age (＞65yrs) | 1.7 | (0.7 - 4) | 0.241 |
|  | History of PCI or CABG | 2.0 | (0.9 - 4.4) | 0.100 |
|  | Heart rate (＞100b.p.m.) | 0.8 | (0.3 - 2.1) | 0.666 |
|  | Fasting blood-glucose | 1.0 | (0.9 - 1.1) | 0.925 |
|  | Renal insufficiency (eGFR <60 mL/min) | 3.0 | (1.4 - 6.5) | 0.005 |
|  | Anemia | 2.0 | (0.9 - 4.2) | 0.074 |
|  | Backward stepwise regression. | | | |
|  | Interleukin-8 | 1.019 | (1.011 - 1.026) | <0.001 |
|  | Renal insufficiency (eGFR <60 mL/min) | 3.3 | (1.7 - 6.5) | <0.001 |
|  | Anemia | 2.5 | (1.2 - 4.9) | 0.009 |
| Interleukin-1β | Enter regression. | | | |
|  | Interleukin-1β | 1.012 | (0.924 - 1.11) | 0.791 |
|  | Age (＞65yrs) | 1.9 | (0.8 - 4.5) | 0.143 |
|  | History of PCI or CABG | 2.6 | (1.1 - 6.1) | 0.023 |
|  | Heart rate (＞100b.p.m.) | 0.9 | (0.3 - 2.6) | 0.911 |
|  | Fasting blood-glucose | 1.1 | (0.9 - 1.2) | 0.268 |
|  | Renal insufficiency (eGFR <60 mL/min) | 2.8 | (1.3 - 6.1) | 0.009 |
|  | Anemia | 1.9 | (0.9 - 4.1) | 0.093 |
|  | Backward stepwise regression. | | | |
|  | Interleukin-1β |  |  | 0.838 |
|  | History of PCI or CABG | 2.9 | (1.3 - 6.2) | 0.007 |
|  | Renal insufficiency (eGFR <60 mL/min) | 3.6 | (1.8 - 7.2) | <0.001 |
|  | Anemia | 2.6 | (1.3 - 5.1) | 0.006 |
| Interleukin-6 | Enter regression | | | |
|  | Interleukin-6 | 1.000 | (0.992 - 1.009) | 0.966 |
|  | Age (＞65yrs) | 1.9 | (0.8 - 4.5) | 0.150 |
|  | History of PCI or CABG | 2.7 | (1.2 - 6) | 0.012 |
|  | Heart rate (＞100b.p.m.) | 1.0 | (0.4 - 2.5) | 0.977 |
|  | Fasting blood-glucose | 1.1 | (0.9 - 1.2) | 0.277 |
|  | Renal insufficiency (eGFR <60 mL/min) | 2.8 | (1.3 - 6.2) | 0.008 |
|  | Anemia | 1.9 | (0.9 - 4.1) | 0.084 |
|  | Backward stepwise regression. | | | |
|  | Interleukin-6 |  |  | 0.824 |
|  | History of PCI or CABG | 2.9 | (1.3 - 6.2) | 0.007 |
|  | Renal insufficiency (eGFR <60 mL/min) | 3.6 | (1.8 - 7.2) | <0.001 |
|  | Anemia | 2.6 | (1.3 - 5.1) | 0.006 |
| Interleukin-10 | Enter regression | | | |
|  | Interleukin-10 | 1.009 | (0.966 - 1.055) | 0.675 |
|  | Age (＞65yrs) | 1.9 | (0.8 - 4.6) | 0.140 |
|  | History of PCI or CABG | 2.6 | (1.1 - 6) | 0.028 |
|  | Heart rate (＞100b.p.m.) | 0.9 | (0.3 - 2.5) | 0.868 |
|  | Fasting blood-glucose | 1.1 | (0.9 - 1.2) | 0.261 |
|  | Renal insufficiency (eGFR <60 mL/min) | 2.8 | (1.3 - 6.1) | 0.010 |
|  | Anemia | 1.9 | (0.9 - 4.1) | 0.102 |
|  | Backward stepwise regression | | | |
|  | Interleukin-10 | / | / | 0.715 |
|  | History of PCI or CABG | 2.9 | (1.3 - 6.2) | 0.007 |
|  | Renal insufficiency (eGFR <60 mL/min) | 3.6 | (1.8 - 7.2) | <0.001 |
|  | Anemia | 2.6 | (1.3 - 5.1) | 0.006 |
| Interleukin | Enter regression | | | |
|  | Interleukin-8 | 1.017 | (1.008 - 1.025) | <0.001 |
|  | Soluble interleukin-2 receptor | 1.000 | (1 - 1.001) | 0.020 |
|  | Interleukin-1β | 1.077 | (0.863 - 1.344) | 0.513 |
|  | Interleukin-6 | 1.003 | (0.997 - 1.009) | 0.276 |
|  | Interleukin-10 | 0.982 | (0.87 - 1.108) | 0.768 |
|  | Backward stepwise regression | | | |
|  | Interleukin-8 | 1.017 | (1.01 - 1.024) | <0.001 |
|  | Soluble interleukin-2 receptor | 1.001 | (1 - 1.001) | 0.014 |

long-term follow-up, a median follow-up of 2.2 years. Abbreviations: CABG, coronary artery bypass grafting surgery; PCI, percutaneous coronary intervention.
