# Supplementary material for: Soluble interleukin-2 receptor combined with interleukin-8 is a powerful predictor of future adverse cardiovascular events in patients with acute myocardial infarction
Source: Front Cardiovasc Med. 2023 Apr 17;10:1110742. doi: 10.3389/fcvm.2023.1110742 (PMC10150071; doi:10.3389/fcvm.2023.1110742)
Supplement: Supplementary file 3 [file Table3.docx]

Supplementary Table 3. Baseline characteristics of the study population stratified by sIL-2R and IL-8 levels

| Factors | All patients N=173 | High sIL-2R^a^  N = 16 | Low sIL-2R^b^  N = 157 | *p* Value | High IL-8^c^  N=38 | Low IL-8^d^ N=135 | *p* Value |
| --- | --- | --- | --- | --- | --- | --- | --- |
| Demographic Information | | | | | | | |
| Age, yrs | 62.9 ±11.4 | 71.6 ±8.7 | 62.1 ±11.3 | <0.001 | 65.5 ±12.1 | 62.2 ±11.2 | 0.103 |
| Male | 147 (85.0) | 12 (75.0) | 135 (86.0) | 0.421^e^ | 32 (84.2) | 115 (85.2) | 0.882 |
| STEMI (vs. NSTEMI) | 93 (53.8) | 9 (56.3) | 84 (53.5) | 0.834 | 24 (63.2) | 69 (51.1) | 0.188 |
| Body mass index, kg/m^2^ | 24.3 (4.5) | 23.3 (4.5) | 24.4 (4.3) | 0.378 | 24.8 (4.7) | 24.2 (4.5) | 0.295 |
| Cardiovascular Risk Factor | | | | | | | |
| Heart rate (＞100b.p.m.) | 21 (12.1) | 5 (31.3) | 16 (10.2) | 0.040^e^ | 7 (18.4) | 14 (10.4) | 0.289^e^ |
| SBP (＜100 mmHg) | 12 (6.9%) | 2 (12.5) | 10 (6.4) | 0.687^e^ | 5 (13.2) | 7 (5.2) | 0.178^e^ |
| Current smoker | 79 (45.7) | 9 (56.3) | 70 (44.6) | 0.372 | 25 (65.8) | 69 (51.1) | 0.109 |
| Diabetes mellitus | 52 (30.1) | 6 (37.5) | 46 (29.3) | 0.693^e^ | 16 (42.1) | 36 (26.7) | 0.067 |
| Hypertension | 106 (61.3) | 14 (87.5) | 92 (58.6) | 0.024 | 20 (52.6) | 86 (63.7) | 0.216 |
| Hypercholesterolemia | 16 (9.2) | 0 (0.0) | 16 (10.2) | 0.375^e^ | 6 (15.8) | 10 (7.4) | 0.208^e^ |
| Coronary artery disease | 103 (59.5) | 9 (56.3) | 94 (59.9) | 0.779 | 30 (78.9) | 73 (54.1) | 0.006 |
| Heart failure | 24 (13.9) | 5 (31.3) | 19 (12.1) | 0.083^e^ | 6 (15.8) | 18 (13.3) | 0.699 |
| History of PCI or CABG | 19 (11.0) | 1 (6.3) | 18 (11.5) | 0.829^e^ | 7 (18.4) | 12 (8.9) | 0.172^e^ |
| History of stroke | 6 (3.5) | 2 (12.5) | 4 (2.5) | 0.097^f^ | 0 (0.0) | 6 (4.4) | 0.412^e^ |
| Renal insufficiency (eGFR <60 mL/min) | 37 (21.4) | 10 (62.5) | 27 (17.2) | <0.001^e^ | 12 (31.6) | 25 (18.5) | 0.083 |
| Anemia | 55 (31.8) | 13 (81.3) | 42 (26.8) | <0.001 | 15 (39.5) | 40 (29.6) | 0.250 |
| Total cholesterol (mg/dL) | 4.41 (1.30) | 3.6 (1.1) | 4.4 (1.3) | 0.002 | 4.64 (1.31) | 4.30 (1.23) | 0.404 |
| HDL cholesterol (mg/dL) | 1.04 (0.34) | 1.1 (0.5) | 1.0 (0.3) | 0.368 | 1.14 (0.36) | 1.01 (0.33) | 0.066 |
| Fasting blood sugar | 6.05 (2.18) | 7.4 (7.1) | 6.0 (2.1) | 0.040 | 6.9 (3.4) | 6.0 (2.0) | 0.005 |
| Biomarkers | | | | | | | |
| cTnT, ng/mL | 2.1 (3.6) | 1.5 (3.9) | 2.2 (3.5) | 0.196 | 2.9 (4.0) | 1.9 (3.6) | 0.331 |
| hs-CRP, mg/dL | 13.2 (34.6) | 65.3 (114.2) | 12.0 (30.4) | <0.001 | 32.0 (59.4) | 12.9 (29.5) | 0.057 |
| NT-proBNP (pg/mL) | 797.6 (1630.0) | 1754.0 (3639.5) | 723.0 (1439.0) | 0.327 | 1699.0 (2754.0) | 679.2 (1222.1) | 0.014 |
| Interleukin cytokines | | | | | | | |
| IL-1β (pg/mL) | 5.0 (0.0) | 1 (6.3) | 10 (6.4) | 1.000^e^ | 7 (18.4) | 4 (3.0) | 0.002^e^ |
| Soluble IL-2 receptor (U/mL) | 399.0 (209) | / | / | / | 5 (13.2) | 11 (8.1) | 0.532^e^ |
| IL-6 (pg/mL) | 12.9 (16.8) | 14 (87.5) | 79 (50.3) | 0.004 | 27 (71.1) | 66 (48.9) | 0.015 |
| IL-8 (pg/mL) | 15.0 (20.5) | 5 (31.3) | 33 (21.0) | 0.532^e^ | / | / | / |
| IL-10 (pg/mL) | 5.0 (0.0) | 6 (37.5) | 20 (12.7) | 0.023^e^ | 14 (36.8) | 12 (8.9) | <0.001 |

^a^ High sIL-2R: greater than the cutoff value 807 U/mL; ^b^ Low sIL-2R: less than or equal to the cutoff value 807 U/mL; ^c^ High IL-8: greater than the cutoff value 32.5 pg/mL; ^d^ Low IL-8: less than or equal to the cutoff value 32.5 pg/mL

Values are median (interquartile range) or % (n). e = Continuity Correction; f = Fisher's Exact Test.

Abbreviations: CABG, coronary artery bypass grafting surgery; cTnT, cardiac troponin T; eGFR, estimated glomerular filtration rate; HDL, high-density lipoprotein; hs-CRP, high-sensitivity C-reactive protein; IL, Interleukin; MACEs, major adverse cardiovascular events; MI, myocardial infarction; NSTEMI, non-ST-segment elevation myocardial infarction; NT-proBNP, N-terminal pro-B-type natriuretic peptide; PCI, percutaneous coronary intervention; STEMI, ST-segment elevation myocardial infarction; SBP, Systolic blood pressure; sIL-2R, soluble IL-2 receptor.
