# Supplementary material for: Soluble interleukin-2 receptor combined with interleukin-8 is a powerful predictor of future adverse cardiovascular events in patients with acute myocardial infarction
Source: Front Cardiovasc Med. 2023 Apr 17;10:1110742. doi: 10.3389/fcvm.2023.1110742 (PMC10150071; doi:10.3389/fcvm.2023.1110742)
Supplement: Supplementary file 5 [file Table5.docx]

Supplementary Table 5. Univariable and multivariable HR of high IL-8 for MACEs after long-term follow-up

|  | HR | 95% Cl. | *p* Value |
| --- | --- | --- | --- |
| Univariable analysis | | | |
| High IL-8 (> Cutoff value) ^a^ | 3.3 | 1.8 - 6.4 | <0.001 |
| Age (> 65yrs） | 1.9 | 0.8 - 4.6 | 0.140 |
| History of PCI or CABG | 2.6 | 1.1 - 6.0 | 0.028 |
| Heart rate (> 100b.p.m.) | 0.9 | 0.3 - 2.5 | 0.868 |
| Fasting blood sugar | 1.1 | 0.9 - 1.2 | 0.261 |
| Renal insufficiency (eGFR <60 mL/min) | 2.8 | 1.3 - 6.1 | 0.010 |
| Anemia | 1.9 | 0.9 - 4.1 | 0.102 |
| Multivariable analysis | | | |
| Model 1^b^ | | | |
| High IL-8 (> Cutoff value) ^a^ | 4.1 | 2.1 - 8.1 | <0.001 |
| Renal insufficiency (eGFR <60 mL/min) | 3.4 | 1.7 - 6.6 | <0.001 |
| Anemia | 3.1 | 1.6 - 6.1 | <0.001 |
| Model 2^c^ | | | |
| High IL-8 (> Cutoff value) | 3.6 | 1.8 - 7.4 | <0.001 |
| Renal insufficiency (eGFR <60 mL/min) | 3.4 | 1.7 - 6.8 | <0.001 |
| Anemia | 3.3 | 1.6 - 6.6 | <0.001 |

^a^ High IL-8 defined as IL-8 levels greater than the cutoff value 32.5 pg/mL. long-term follow-up, a median follow-up of 2.2 years.

^b^ Model 1 adjusted for age (＞65yrs), history of PCI or CABG, heart rate (＞100b.p.m.), fasting blood sugar, renal insufficiency (eGFR <60 mL/min), anemia.

^c^ Model 2: model 1 + sex, body mass index, current smoker, diabetes mellitus, hypertension, hypercholesterolemia, coronary artery disease, heart failure, history of PCI or CABG, history of stroke, total cholesterol, HDL cholesterol, and fasting blood sugar.

Abbreviations: CABG, coronary artery bypass grafting surgery; HDL, high-density lipoprotein; PCI, percutaneous coronary intervention.
