# Supplementary material for: Soluble interleukin-2 receptor combined with interleukin-8 is a powerful predictor of future adverse cardiovascular events in patients with acute myocardial infarction
Source: Front Cardiovasc Med. 2023 Apr 17;10:1110742. doi: 10.3389/fcvm.2023.1110742 (PMC10150071; doi:10.3389/fcvm.2023.1110742)
Supplement: Supplementary file 6 [file Table6.docx]

Supplementary Table 6. AIC and BIC results of the model.

| Models | AIC | BIC |
| --- | --- | --- |
| GRACE score | 183.939 | 439.356 |
| sIL-2R | 183.939 | 439.356 |
| IL-8 | 171.213 | 410.864 |
| sIL-2R+IL-8 | 345.386 | 887.752 |
| GRACE score+sIL-2R | 346.000 | 891.519 |
| GRACE score+IL-8 | 334.000 | 860.600 |
| GRACE scoresIL-2R+IL-8 | 346.000 | 891.519 |

Abbreviations: AIC, Akaike Information Criterion; BIC, Bayesian Information criterion; GRACE, Global Registry of Acute Coronary Events; IL, interleukin; sIL-2R, soluble IL-2 receptor.
