# Supplementary figures and images for: Soluble interleukin-2 receptor combined with interleukin-8 is a powerful predictor of future adverse cardiovascular events in patients with acute myocardial infarction
Source: Front Cardiovasc Med. 2023 Apr 17;10:1110742. doi: 10.3389/fcvm.2023.1110742 (PMC10150071; doi:10.3389/fcvm.2023.1110742)

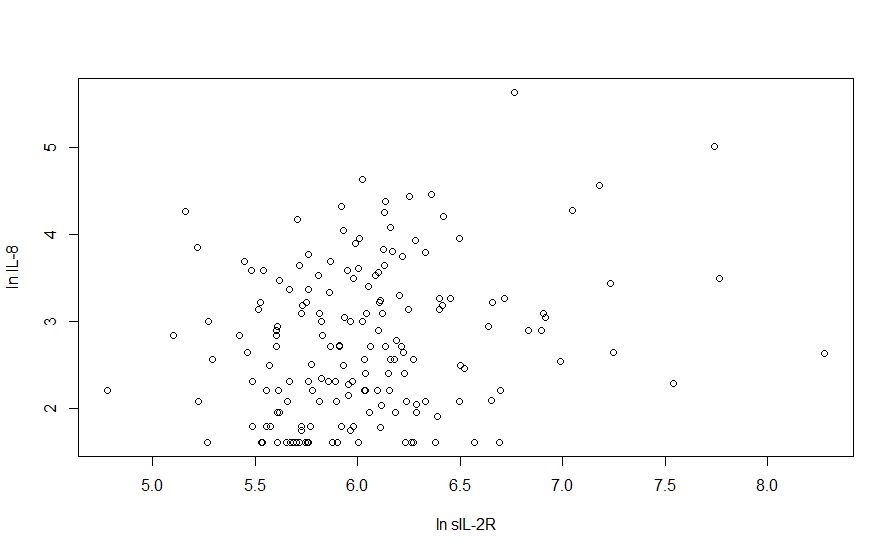

Supplement: Supplementary file 7 [file Image1.tiff]

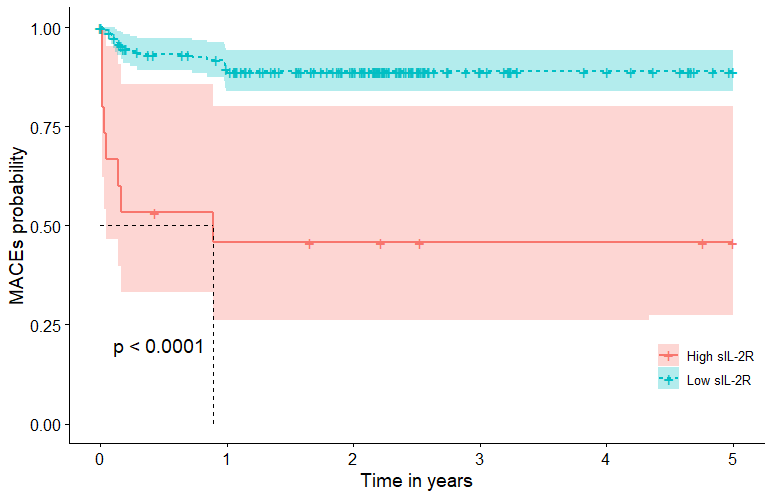

Supplement: Supplementary file 8 [file Image2.tiff]

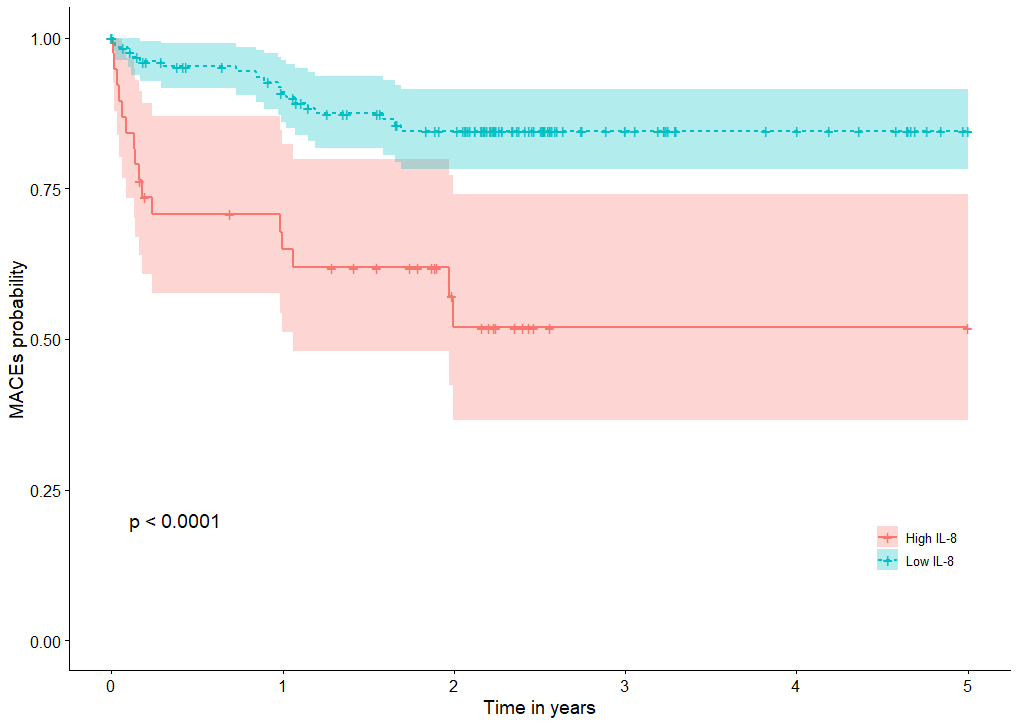

Supplement: Supplementary file 9 [file Image3.tiff]

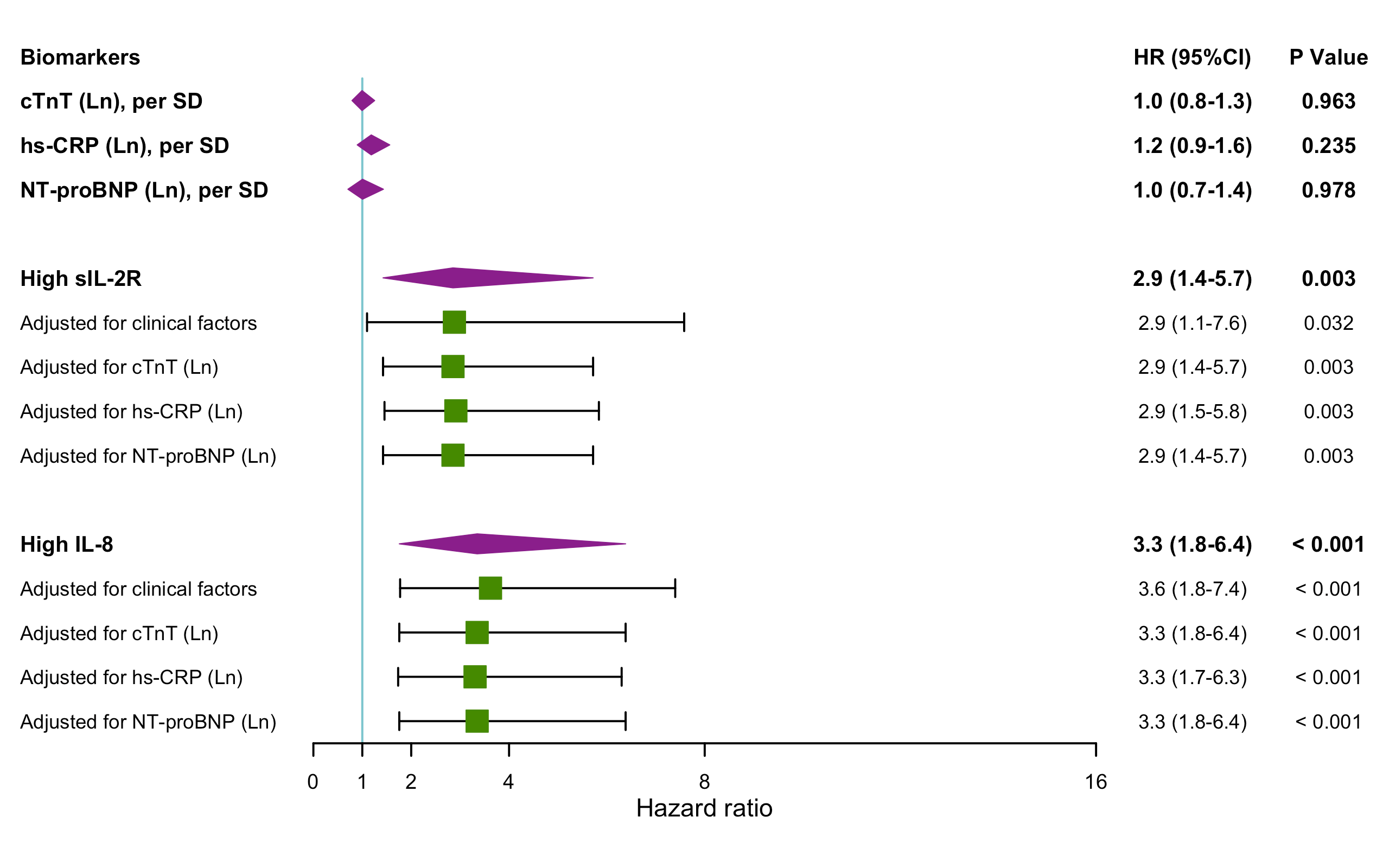

Supplement: Supplementary file 10 [file Image4.tiff]

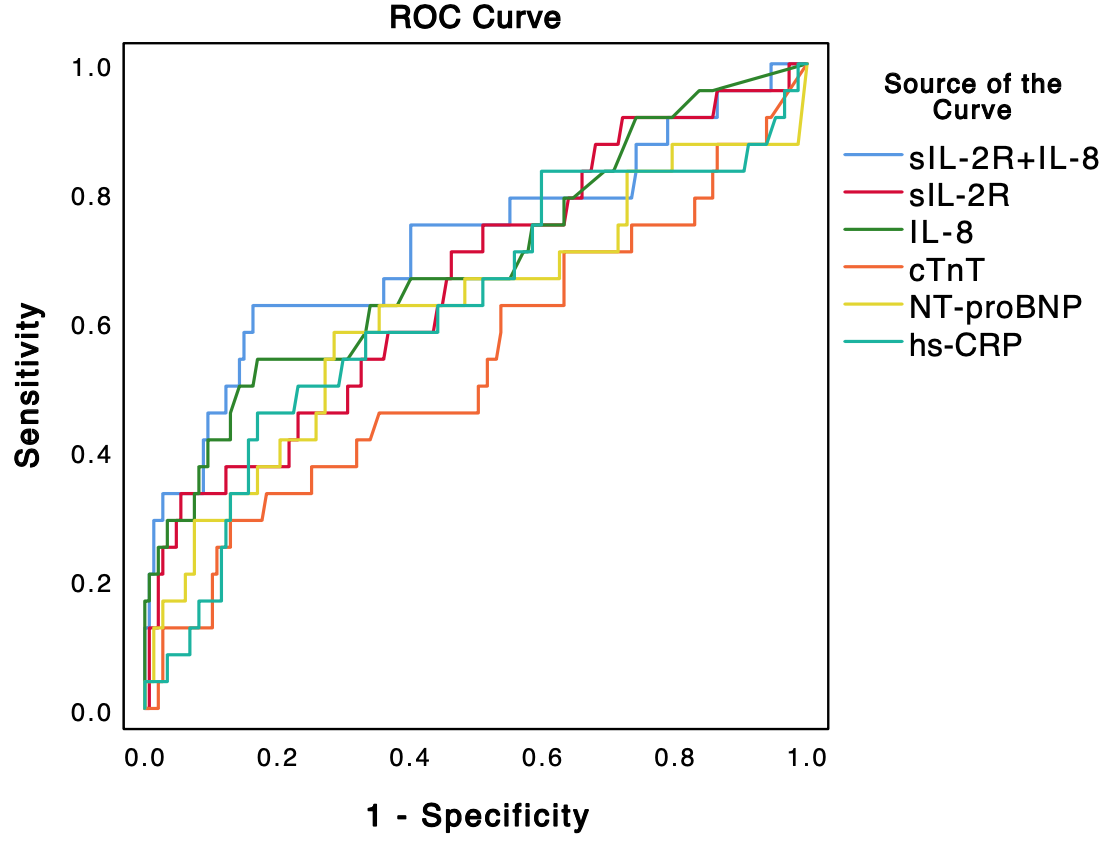

Supplement: Supplementary file 11 [file Image5.tiff]
